# A molecular mechanism for transthyretin amyloidogenesis

**DOI:** 10.1038/s41467-019-08609-z

**Published:** 2019-02-25

**Authors:** Ai Woon Yee, Matteo Aldeghi, Matthew P. Blakeley, Andreas Ostermann, Philippe J. Mas, Martine Moulin, Daniele de Sanctis, Matthew W. Bowler, Christoph Mueller-Dieckmann, Edward P. Mitchell, Michael Haertlein, Bert L. de Groot, Elisabetta Boeri Erba, V. Trevor Forsyth

**Affiliations:** 1 Faculty of Natural Sciences, Keele University, Staffordshire, ST5 5BG UK; 2 Institut Laue-Langevin, 71 avenue des Martyrs, 38042 Cedex 9 Grenoble, France; 3 Max Planck Institute for Biophysical Chemistry, Am Faßberg 11, 37077 Göttingen, Germany; 4 Heinz Maier-Leibnitz Zentrum (MLZ), Technische Universität München, 85748 Garching, Germany; 5 Univ. Grenoble Alpes, CEA, CNRS, IBS, 38000 Grenoble, France; 6 European Synchrotron Radiation Facility, 71 avenue des Martyrs, 38042 Cedex 9 Grenoble, France; 7 EMBL, Grenoble Outstation, 71 avenue des Martyrs, 38042 Cedex 9 Grenoble, France

## Abstract

Human transthyretin (TTR) is implicated in several fatal forms of amyloidosis. Many mutations of TTR have been identified; most of these are pathogenic, but some offer protective effects. The molecular basis underlying the vastly different fibrillation behaviours of these TTR mutants is poorly understood. Here, on the basis of neutron crystallography, native mass spectrometry and modelling studies, we propose a mechanism whereby TTR can form amyloid fibrils via a parallel equilibrium of partially unfolded species that proceeds in favour of the amyloidogenic forms of TTR. It is suggested that unfolding events within the TTR monomer originate at the C-D loop of the protein, and that destabilising mutations in this region enhance the rate of TTR fibrillation. Furthermore, it is proposed that the binding of small molecule drugs to TTR stabilises non-amyloidogenic states of TTR in a manner similar to that occurring for the protective mutants of the protein.

## Abstract (web summary)

A number of disease-causing human transthyretin (TTR) mutations are known to lead to amyloid formation. Here the authors combine neutron crystallography, native mass spectrometry and modelling studies to characterize the T119M and S52P-TTR mutants, providing mechanistic insights into TTR amyloidosis.

## Introduction

**Fig. 1:**
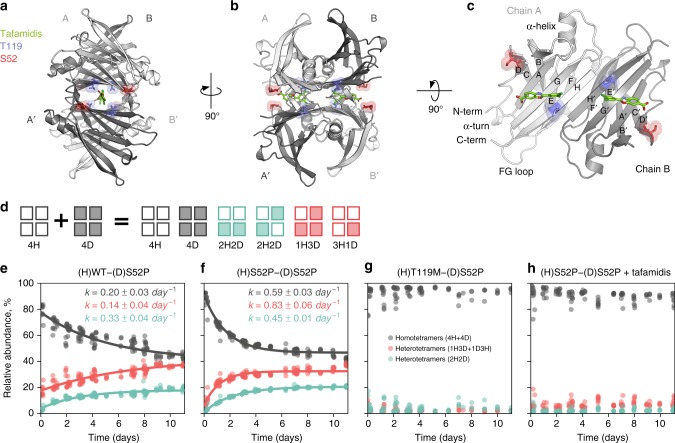
Structure of human TTR and experimental studies of TTR stability. **a**–**c** Crystallographic structure of WT-TTR (PDB-ID 4PVM). Tafamidis is shown in green (by superimposition of PDB-ID 6FFT), the S52 amino acid in red, and T119 in blue. **d** Scheme of the native MS experiment: 4H (hydrogenated) and 4D (deuterated) tetramers are mixed in equal parts; following dissociation, four hybrid tetrameric species may form by the assembly of monomeric and dimeric species. **e**–**h** MS result for the subunit exchange between deuterated S52P and hydrogenated WT, S52P, T119M, and S52P bound to tafamidis, respectively. Changes in relative abundance of homo- and hetero-tetrameric species over the course of 11 days are shown, along with an estimate of the of their association/dissociation rates (uncertainties are one standard deviation of the posterior densities)

Human transthyretin (TTR) is a 55 kDa homotetrameric protein that transports thyroxine (T_4_) and retinol-binding protein (RBP) in the serum and cerebrospinal fluid (Fig. 1a–c). Wild-type (WT) TTR is intrinsically amyloidogenic, and causes amyloid fibril formation in elderly individuals, resulting in senile systemic amyloidosis (SSA)^1,2^. A large number ( > 100) of TTR mutants have been identified (http://amyloidosismutations.com). The majority of these are implicated in familial amyloid polyneuropathy/myocardiopathy (FAP/FAC) and several other forms of amyloidosis^3–5^. Of special interest are the T119M and S52P mutations. The T119M mutation is non-amyloidogenic, and heterozygous individuals carrying the T119M mutation and an amyloidogenic mutation, such as V30M, either remain asymptomatic of FAP or present a more benign form of the disease^6,7^. On the other hand, individuals carrying the S52P mutation develop aggressive and early-onset fatal amyloidosis^8,9^. Currently, only one drug—tafamidis (trade name *Vyndaqel*; Pfizer)—has been granted market authorisation for the treatment of TTR-FAP in adult patients having stage 1 symptomatic polyneuropathy^10^.

Considerable effort has been invested in seeking to identify the factors that underlie TTR amyloid formation including X-ray diffraction^11–13^, NMR^14–16^, solution scattering^17^, and mass spectrometry (MS)^18^. However, a molecular level understanding of the widely different behaviours of various TTR mutants has remained elusive^11,12^. In this paper, native MS, neutron crystallography, and computer simulations have been used to investigate the effects of the T119M and S52P mutations on TTR stability and the molecular determinants of amyloid formation. It is found that the S52P mutation destabilises TTR by altering the stability of the CD loop in the protein monomer. In contrast, the T119M mutation stabilises the dimer–dimer interface as well as TTR’s tertiary structure. Furthermore, it is shown how the stability of TTR tetramers is coupled to those of the monomers and dimers. Finally, it is suggested that tafamidis stabilises native tetrameric TTR in a similar way to that occurring for the T119M mutant in terms of thermodynamic, kinetic and structural features. A molecular mechanism is proposed by which the vastly different behaviours of the various TTR mutants can be understood.

## Results

### Kinetics of TTR tetramer dissociation

Tetramer dissociation is thought to be the rate-limiting step in amyloid formation^19–22^. MS was used to follow the subunit exchange dynamics of TTR over several days so that the effect of the two mutations, and that of tafamidis, on the rate of dissociation of the tetramer species could be compared. These experiments relied on the use of hydrogenated (H) and deuterated (D) TTR to track otherwise identical protein chains. H- and D-TTR were mixed in an equimolar ratio and monitored over time to observe the rate of dissociation of the homo-tetramers (4H, 4D) and the rate of formation of the hetero-tetramers (2H2D, 1H3D, 3H1D; Fig. 1d and Supplementary Figure 1). The two 2H2D assemblies that can be formed may have different association constants, given that one requires only association of homo-dimers while the other requires the formation of hetero-dimers before assembly into tetramers. However, these two forms cannot be differentiated by MS, and are necessarily considered together. Since it has been shown that the kinetics of TTR subunit exchange is susceptible to isotope effects^18^, a deuterated TTR species [(D)S52P] was used as a reference to allow a meaningful comparison of the TTR variants. The change in relative abundance of each tetrameric species over time, and the relative association/dissociation rate constants are shown in Fig. 1e–h.

The dissociation rate of homo-tetramers in the (H)WT-(D)S52P experiment (Fig. 1e) was 0.20 ± 0.03 day^−1^. Among the hetero-tetramer species, those containing identical dimers (2H2D; two hydrogenated and two deuterated TTR monomers) were formed at a higher rate (0.33 ± 0.04 day^−1^) than those formed by two different dimers (3H1D and 1H3D; 0.14 ± 0.03 day^−1^). The observed rate of dissociation of homo-tetramers in the (H)S52P-(D)S52P experiment (Fig. 1f) was about three times larger than in the (H)WT-(D)S52P (0.59 ± 0.03 day^−1^). In this case, however, the 3H1D/1H3D species were formed faster than the 2H2D ones: 0.83 ± 0.06 day^−1^ and 0.45 ± 0.01 day^−1^, respectively. In the (H)T119M-(D)S52P experiment (Fig. 1g), almost no tetramer dissociation was observed over the course of eleven days—a timescale over which essentially all of the TTR would be recycled in a physiological context (the biological half-life of TTR is 1–2 days^23^). A final experiment was carried out with (H)S52P and (D)S52P in the presence of tafamidis at a molar ratio of 1:1 (Fig. 1h). In this case, tetramer dissociation was almost eliminated by the presence of the drug. This also demonstrated that one molecule of tafamidis per tetramer was sufficient for effective stabilisation.

Overall, the MS experiments revealed that the S52P mutation increases the rate of dissociation of TTR tetramers by about a factor of three as compared with the WT (reduced tetramer stability was also observed as a function of pH—see Supplementary Figure 2a-b). In strong contrast, both the T119M mutation and tafamidis effectively abolished tetramer dissociation.

### Effect of mutations on TTR equilibria

**Fig. 2:**
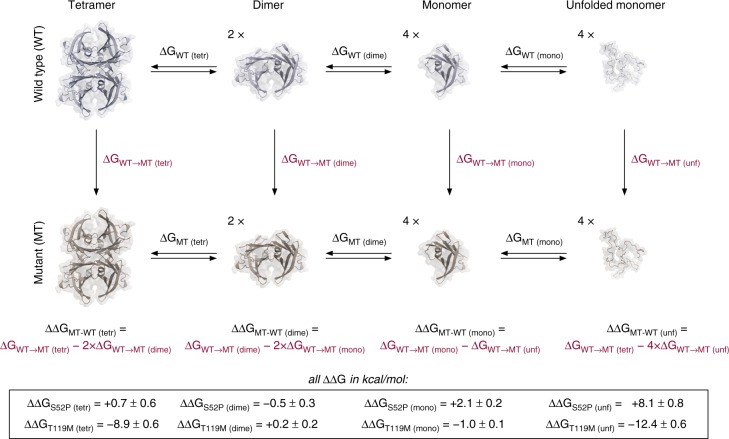
Relative stability of mutant tetramers, dimers, and monomers, with respect to WT-TTR. ΔΔG_(tetr)_ is the stability of the mutant tetramers with respect to WT-TTR for the process of dissociation to dimers; ΔΔG_(dime)_ represents the relative stability of the mutant dimers when they dissociate to monomers; ΔΔG_(mono)_ indicates the relative stability of the mutant monomers for unfolding; ΔΔG_(unf)_ is the relative stability of the mutant tetramers for the overall processes of tetramer dissociation and unfolding. The uncertainties shown are one standard error of the mean, obtained from ten repeated calculations

Free energy (G) calculations were used to study the effects of the T119M and S52P mutations on the thermodynamic stability of TTR tetramers, dimers and monomers. These calculations were based on MD simulations^24^ and the thermodynamic cycle is shown in Fig. 2. Control over the potential energy function used allowed specific residues to be changed into other ones, so that the free energy change associated with the modifications could be calculated for the protein in its tetrameric (ΔG_tetr_), dimeric (ΔG_dime_) or monomeric (ΔG_mono_) states. Differences between these ΔG values provide an estimate of the stability of the mutant tetramers, dimers and monomers relative to the WT: ΔΔG_tetr_,_,_ ΔΔG_dime_, and ΔΔG_mono_ respectively (Fig. 2).

The calculations suggested that the S52P mutation would not have a significant effect on the stability of the tetramers and dimers for their dissociation to dimers and monomers, respectively (ΔΔG_tetr_ and ΔΔG_dime_). However, a large and statistically significant ΔΔG_mono_ ( + 2.1 kcal mol^−1^) indicated that the introduction of the S52P mutation had a destabilising effect on the monomer with respect to the WT-TTR, corresponding to a shift towards unfolded TTR by a factor of ~34. The T119M mutation had no effect on the stability of the dimers. However, in contrast to the case for S52P, a large stabilising effect on the tetramers was observed (ΔΔG_tetr_ = −8.9 kcal mol^−1^; equilibrium constant fold-change of ~3∙10^6^). In addition, the T119M monomers were calculated to be more stable than WT monomers by 1 kcal mol^−1^ (~5-fold increase in stability), a small but statistically significant effect.

When considering the overall process of tetramer dissociation and unfolding, the S52P mutation was estimated to shift the equilibrium toward the unfolded state by 8.1 kcal mol^−1^ (a change of ~8∙10^5^), whereas the T119M mutation was estimated to have the opposite effect of stabilising the tetramers by 12.4 kcal mol^−1^ (a change of ~10^9^). Given that the two mutation sites are not in close contact, it is reasonable to assume additivity for their associated energetic effects and on this basis the data suggest that in a double S52P/T119M-TTR mutant, the T119M mutation would be able to balance the destabilising effect of the S52P mutation; this is in agreement with the observed in vivo protective effect of the T119M mutation^25,26^.

In summary, the free energy calculations show that the S52P mutation results in a TTR protein having a less stable fold. However, this appears to have little direct impact on tetramer and dimer dissociation. In contrast, the T119M mutation directly affects tetramer dissociation to dimers by stabilising the tetramer species and also has the effect of stabilising the protein fold.

### Neutron diffraction reveals the basis of TTR instability

In order to study the intramolecular interactions of S52P- and T119M-TTR in atomic detail, room-temperature neutron crystallography studies were carried out. The crystal structures of the S52P mutant, the T119M mutant, and the S52P mutant in complex with tafamidis, were determined at resolutions of 1.80 Å, 1.85 Å, and 2.00 Å, respectively. Most of the interaction network at the monomer–monomer and dimer–dimer interfaces (Supplementary Figure 3), as well as the energetics of bridging water molecules (Supplementary Figure 4), were found to be conserved across the WT and mutant structures. However, crucial differences are identified as the source of the different kinetic and thermodynamic stabilities observed.

In the T119M structure, the longer Met119 side chain extends across the thyroxine-binding channel into a hydrophobic pocket surrounded by residues Leu17, Ala19, Leu110 and Val121 (Supplementary Figure 5). These interactions, as also noted in previous studies, enhance the association of two dimers and hence the overall stability of the tetramer^27^.

**Fig. 3:**
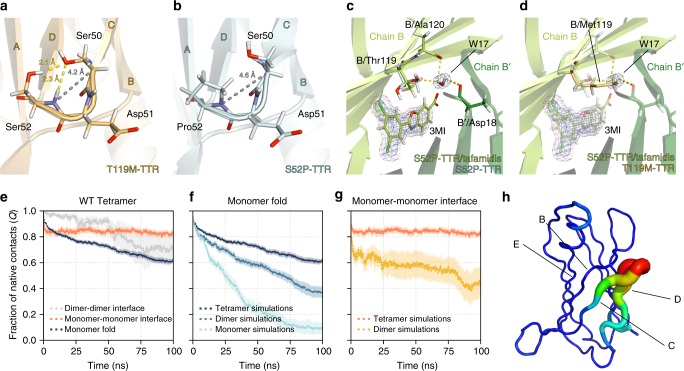
Neutron structures and molecular dynamics results. **a** In the T119M mutant (as well as in the WT; not shown), the Ser52 residue forms two hydrogen bonds (*yellow dash lines*) with the Ser50 residue. The distance between Ser50-C_α_ and the amide-N of Ser52 is 4.2 Å (grey), and it is the same in the WT (not shown). **b** In the S52P mutant, due to the absence of the  hydrogen bonds between Pro52 and Ser50, the distances between Ser50-C_α_ and the amide-N of Pro52 is longer (4.6 Å), creating a looser CD loop. **c** Binding of tafamidis (3MI) results in a change of orientation for residue Thr119 (cyan, before binding; green, after binding). In the S52P/tafamidis complex, the hydroxyl side chain of Thr119 forms a hydrogen bond to a water molecule. **d** The water molecule in the S52P/tafamidis complex occupies the same position as the side chain of Met119 residue (light orange) in the non-pathogenic T119M mutant. The binding site between chain B and B′ is shown. The blue and magenta mesh show the 2*F*_*o*_*-F*_*c*_ neutron scattering length density map and the 2*F*_*o*_*-F*_*c*_ X-ray electron density map, respectively. Maps were contoured at 1.5 σ. **e**–**g** Loss of native fold and contacts in WT-TTR during high-temperature MD simulations with the Amber99sb*-ILDNP force field; shown are the mean and its standard error from 10 simulation repeats (results for Charmm36 are in Supplementary Figure 6). **e** Degree of disruption of the monomer fold, the monomer–monomer interface, and the dimer–dimer interface, when simulating the WT tetramer over the course of 100 ns. **f** Degree of unfolding of TTR monomers as part of a tetramer, dimer, or monomer in solution. **g** Degree of disruption of the monomer–monomer interface when simulating the dimer and the tetramer. **h** Protein regions in the PLS-FMA mode contributing most to the change in the fraction of native contacts for the high-temperature simulations

The amino acid at position 52 plays a crucial role. In the WT and the T119M mutant structures, the amide-D and the side-chain hydroxyl of the Ser52 residue form hydrogen bonds with O_Ɣ_ of Ser50, resulting in a stable CD loop (Fig. 3a). The highly amyloidogenic S52P mutant has a proline residue at position 52 in place of the serine present in the WT. The lack of a main-chain amide-H and a side-chain hydroxyl group in the proline residue prevents the formation of two hydrogen bonds and causes a loss of stability of the protein (Fig. 3b). The distance between amide-N of residue 52 and C_α_ of Ser50 in the WT and T119M structures is 4.2 Å (Fig. 3a). The same distance is 0.4 Å wider in the S52P mutant, implying a looser CD loop (Fig. 3b). This destabilises the β-turn where the residue is located as well as the associated C and D strands.

In the S52P/tafamidis complex, the CD loop is not stabilised by tafamidis binding and the distance between Ser52-N and Ser50-C_α_ remains as 4.6 Å. In contrast to the findings of Bulawa et al.^28^, no water molecules were found bridging the protein and tafamidis in this complex. Instead, tafamidis induced a 180° flip to the Thr119 side chain (Fig. 3c), with the hydroxyl moiety now forming a hydrogen bond to a water molecule that bridges the two TTR dimers via Asp18; this was observed in both binding sites. Interestingly, this water molecule fills the same space that is occupied by the Met119 side chain in the non-pathogenic T119M mutant (Fig. 3d). Grand canonical Monte Carlo calculations^29^ suggested that the stabilisation of this water molecule upon tafamidis binding may substantially contribute toward the affinity of tafamidis, by about −2.8 kcal mol^−1^ (Supplementary Figure 4).

Collectively, the neutron structures of T119M-TTR, and S52P-TTR in *apo* and *holo* forms, have revealed that most interactions between the β-strands forming the core of the protein are conserved. However, the β-turn between the C and D strands is loosened in S52P-TTR, suggesting that this location is crucial to the reduced stability of this mutant. The appearance of a bridging water molecule in the S52P/tafamidis complex results in a conformation that resembles that of Met119 in T119M-TTR in this region of the protein,  and may provide insight to the  mechanism by which the drug stabilises the TTR tetramer.

### Coupling between quaternary and tertiary structure stability

High-temperature MD simulations were used to study the effect of TTR quaternary structure on the kinetics of TTR unfolding. Simulations were carried out at 598 K for the tetramers, dimers and monomers of WT-TTR with two modern force fields (Amber99sb*-ILDNP^30,31^ and Charmm36^32^). As a measure of unfolding, the fraction of native contacts *Q* was employed^33^, where 1 indicates a folded protein and 0 an unfolded protein. A similar metric was used to define the fraction of monomer–monomer and dimer–dimer interface contacts retained during the high-temperature simulations (Fig. 3e; Charmm36 results in Supplementary Figure 6). At the temperature of 298 K, all monomers and interfaces were stable with *Q* close to one for simulations of up to 1 μs (Supplementary Figure 7).

In Fig. 3f, the degree of unfolding of TTR monomers over the course of 100 ns simulations is shown for simulations of the tetramer, dimer and monomer in solution. The monomer in solution was found to unfold faster than the monomers that were part of a dimer, which in turn unfolded faster than the monomers that were part of the tetramer. Thus, it appeared that the quaternary structure of TTR had an effect on the stabilisation of the individual monomeric chains. It is conceivable that the opposite would hold true as well: i.e., that highly stable monomeric chains (as in T119M) may enhance the stability of the tetramer, whereas unstable monomeric chains (as in S52P) are likely to have a detrimental effect on the stability of the tetramers. The same effect was observed for the stability of the monomer–monomer interface. This interface was more resistant to disruption than the dimer–dimer interface (Fig. 3e). However, when simulating the TTR dimer, the same interface became much more easily disrupted within the timeframe of the simulations (Fig. 3g).

Partial-least squares (PLS) functional mode analysis (FMA)^34,35^ was used to identify regions of the TTR protein that contributed most to its unfolding while being part of a tetrameric unit. In Fig. 3h, a WT-TTR monomer is shown and colour-coded according to the root mean square fluctuations of the maximally correlated mode. The figure shows how the largest loss of native contacts observed was due to large motions in the C and D strands (Charmm36 results in Supplementary Figure 8). Similar observations were also made when calculating the fraction of native contacts by strand (Supplementary Figure 9).

These MD simulations showed that TTR tertiary structure is stabilised by the formation of tetrameric and dimeric units. Similarly, the monomer–monomer interface is stabilised by the association of TTR dimers into tetramers. Taken together, the observations explain why tetramer dissociation is the rate-limiting step in the formation of amyloid. In addition, in agreement with previous NMR data^16,36^, PLS-FMA identify the C and D strands as the regions of TTR tetramers most prone to unfolding.

## Discussion

**Fig. 4:**
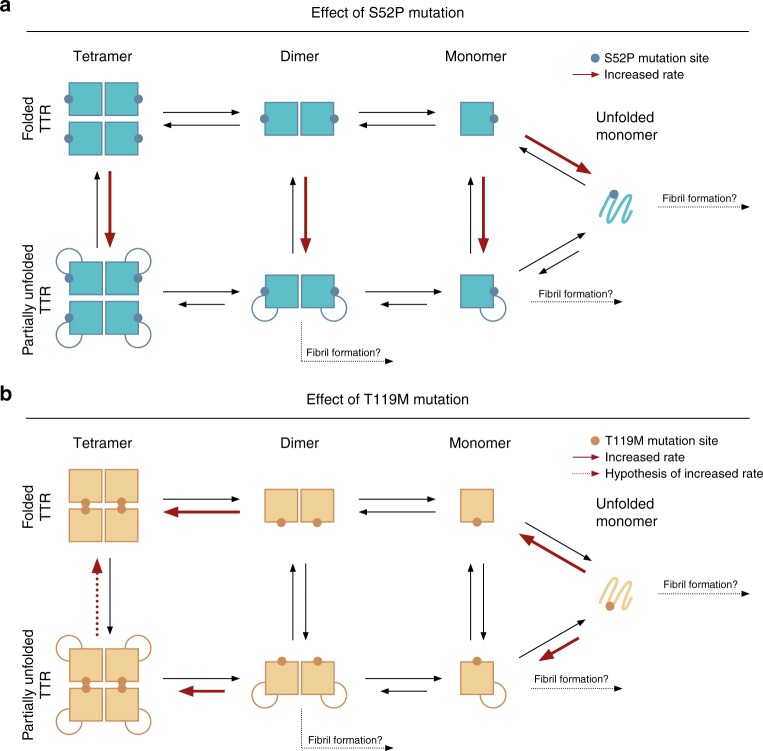
Proposed model of mutational effect on TTR stability. Shorter arrows indicate a lower propensity to association/folding. Red arrows reflect the qualitative change in reaction rates for the mutants with respect to WT-TTR. **a** The S52P mutation increases the likelihood of partial and full unfolding of TTR monomeric units. This, in turn, also leads to a lower stability of TTR multimeric assemblies; hence partially unfolded dimers and monomers, and fully unfolded monomers, are generated at a higher rate. Some or all of these species are then removed from solution via the formation of amyloid fibrils. **b** In contrast to S52P, the T119M mutation stabilises the folded monomeric forms of TTR. Most importantly, this mutation has a large stabilising effect on TTR tetramers, which are believed not to form fibrils. Furthermore, it is proposed that the higher stability of the tetramer also has the effect of further stabilising the monomer fold

Based on the data presented, a molecular mechanism that explains the different behaviours of the T119M and S52P mutants is proposed (Fig. 4). It is suggested that partial unfolding events originating at the CD strands lead to a parallel equilibrium of folded and partially unfolded TTR states (tetramers, dimers and monomers) that allows the effects of the S52P and T119M mutations to be rationalised in the context of the current and previous experimental observations^13,16,36,37^. Given that a number of other pathogenic mutations are located on the C and D strands (e.g. L55P, S50R, E54G), the mechanism proposed here may apply to other mutations in this region of the protein^8,13,38,39^.

S52P-TTR tetramers were observed to dissociate faster than the WT in solution. However, no thermodynamic or structural cause for a direct destabilisation of S52P tetramers could be established from these observations or from those published previously. In contrast, the free energy calculations described here provide evidence that the tertiary structure of S52P-TTR is thermodynamically less stable than that of the WT. This, together with structural (neutron diffraction) and dynamical (simulation) evidence of a loose CD loop, suggests that unfolding events at the CD strands are likely. As it is expected that a partially unfolded TTR would proceed towards dissociated species and amyloid formation faster than the corresponding fully folded protein (Fig. 4a), this parallel equilibrium can explain the high amount of amyloid fibrillation of S52P-TTR (Supplementary Figure 2c) as well as the faster tetramer dissociation rates observed.

T119M-TTR tetramers were found to be highly resistant to dissociation. The free energy calculations estimated a stabilising effect of 8.9 kcal mol^−1^ on the tetramer, which corresponds to a change in equilibrium constant of ~3∙10^6^. This effect can be understood in terms of the increased contact area between the two dimers, which stabilises the dimer–dimer interface (Supplementary Figure 5). In addition, the free energy calculation results suggest a tertiary fold in T119M-TTR that is more stable than in the WT by about 1 kcal mol^−1^. As illustrated in Fig. 4b, tetramer stabilisation directly reduces the population of amyloidogenic building blocks. Furthermore, a stable tertiary structure can protect against the unfolding of monomeric TTR. Finally, the coupling between TTR quaternary structure and the stability of the TTR fold suggested by the simulations indicates that the highly stable T119M-TTR tetramer reduces the probability of the CD strands unfolding (Fig. 4b).

Stabilisation of the native tetrameric structure by small molecule binding to the T4 binding sites of TTR is a well-cited rationale for the inhibition of TTR amyloidogenesis^40^. MS kinetic experiments have shown how tafamidis, like the T119M mutation, strongly inhibits tetramer dissociation. The binding affinity of tafamidis to TTR quantifies the stabilising effect of the drug on TTR tetramers, and was measured to be ~12 kcal mol^−1^ (K_*d*1_ = 2 nM)^28^. The stabilising effect of T119M on the tetramer (with respect to the dimer) was found to be about 9 kcal/mol. However, considering the further gains in TTR fold stability conferred by the mutation (ΔΔG_T119M (mono)_), the overall thermodynamic effect is very similar to that of the tafamidis-S52P complex, with ΔΔG_T119M (unf)_ ≈ −12 kcal mol^−1^. Tafamidis therefore stabilises native TTR thermodynamically and kinetically in a manner that is comparable to T119M. However, it is difficult to predict the extent to which the two separate effects of tetramer and fold stabilisation may impact on the in vivo protection against amyloidogenesis. Structurally, the binding of tafamidis results in the reorientation of Thr119 and the emergence of a water molecule in the binding pocket. This water molecule is located in the same position as the side chain of Met119 in the T119M mutant.

The results presented also suggest that TTR tetramers have a stabilising effect on the monomer–monomer interface, which becomes weakened when TTR is in its dimeric form (Fig. 3g). Thus, even though the monomer–monomer interface is more stable than the dimer–dimer interface^16,41^ when the tetramer is intact, the loss of native quaternary structure can destabilise the monomer–monomer interface such that TTR dimers are able to quickly proceed to further dissociation into monomers (in agreement with the data described here and elsewhere^22,42^). This observation further explains why tetramer dissociation is the rate-limiting step in the formation of amyloid. It is noted that dissociation might not be the only factor causing the formation of TTR fibrils (e.g. seeding with ex vivo fibrils has been found to promote the formation of fibrils in vitro) but it is a necessary one^43^.

Finally, a number of studies^44–47^ have identified a TTR fragment (residues 49–127) in ex vivo amyloid fibrils. This fragment is formed by proteolytic cleavage of the peptide bond between Lys48 and Thr49, with the S52P mutant being particularly susceptible to it^45,47^. Within the mechanism set out in Fig. 4, the higher rate of proteolytic cleavage in S52P-TTR can be explained by an increased accessibility to Lys48 due to a higher propensity to unfold at the CD strands. Cleavage of the unfolded structure causes an irreversible transition to a partially unfolded state, thus preventing refolding and ultimately enhancing the rate of fibril formation.

In summary, the results described here provide novel structural and dynamical insights into the opposing effects of the S52P and T119M mutations in TTR, as well as the effects of tafamidis binding on the stability of TTR. The results provide molecular level detail of direct relevance to TTR amyloidogenicity, and have provided a framework for further investigation into the effects of residue mutation on TTR’s states and their equilibria, and for the development of novel targeted therapies for FAP and SSA.

## Methods

### Protein preparation and crystallization

The cDNA corresponding to the gene coding for the 127 amino acids of human transthyretin (TTR) protein was cloned into a pET-M11 vector encoding an N-terminal His_6_ tag and a TEV cleavage site (EMBL Protein and Purification Facility, Germany) and then expressed in *E.coli* BL21 (DE3) cells (Invitrogen). The S52P and T119M mutants were generated using QuikChange Lightning Multi Site-Directed Mutagenesis kit (Strategene). WT-TTR cDNA sequence and the sequences of primers used for S52P and T119M mutations are shown in Supplementary Table 1. Deuterated protein was produced in the Deuteration Laboratory (D-Lab) platform within ILL's Life Sciences Group^48^. Cells were adapted to perdeuterated Enfors minimal medium and grown in a fed-batch fermenter culture at 30 °C using *d*_8_-glycerol (99% deuterium; Euriso-top) as the only carbon source. H-TTRs and D-TTRs were purified in an identical manner. Cell paste was resuspended homogeneously in lysis buffer (20 mM Tris pH 8, 250 mM NaCl, 3 mM imidazole) in the presence of EDTA-free protease inhibitor cocktail (Roche). The cells were lysed by ultrasonication on ice at 50% amplitude and 25 sec pulses. The lysate was cleared by centrifugation at 18,000 rpm at 4 °C for 30 min. The supernatant containing crude protein extracts was recovered and purified using benchtop gravity-flow chromatography. Nickel-nitriloacetic acid (Ni^2+^-NTA) resin was pre-washed with lysis buffer and left in incubation with the protein at 4 °C for at least an hour to increase binding efficiency. The protein/resin mix was then loaded into 10 ml disposable column. The column was washed three times with wash buffer (20 mM Tris pH 8, 15 mM imidazole, NaCl at 500 mM, 1 M, and 250 mM, respectively) before the protein was eluted (elution buffer: 20 mM Tris pH8, 250 mM NaCl, 250 mM imidazole). Fractions containing protein were then pooled together for TEV protease treatment to remove His_6_-tag and dialysed into gel filtration buffer (10 mM Tris pH 7.5, 50 mM NaCl, 1 mM DTT) overnight at 4 °C. Protein was separated from the cleaved poly-histidine tail using the same gravity-flow column described above. Flowthrough from the column was concentrated, filtered (with 0.22 μm membrane pore size) and loaded onto Superdex 75 HiLoad 16/600 gel filtration column (GE Healthcare) running at 1 ml/min at room temperature. Peak fractions were pooled and concentrated using Amicon Ultra centrifugal filter units (Millipore) and stored at −20 °C. Analysis was carried out on 12% Tris-Tricine gel after each step of purification to monitor the purity of the protein. The details of the expression and purification have been described previously^18,49^. While the deuterated TTR was expressed under conditions where only deuterium atoms were present, protiated (^1^H-based) solutions were used during purification. Prior to protein crystallization, the purified protein was buffer-exchanged using a deuterated solution so that the labile protium atoms acquired during the purification steps that involved hydrogenated solutions were replaced by deuterium. Tafamidis (HPLC purity: ≥ 98%; Carbosynth Limited, U.K.) was dissolved in 100% deuterated dimethyl sulfoxide (D-DMSO) for crystallization. All crystals were grown by sitting-drop vapour diffusion at 18 °C using deuterated protein and deuterated solution. The TTR S52P mutant crystal (~0.60 mm^3^) were grown in 1.9 M malonate pD 6.4 with a protein concentration of 25 mg/ml (drop volume 50 µl; protein-to-buffer ratio of 7:5), while the T119M mutant crystal (~0.80 mm^3^) were in 1.9 M malonate pD 5.9 with 40 mg/ml protein concentration (drop volume 50 µl; protein-to-buffer ratio of 1:1). For the crystal of the S52P/tafamidis complex (~0.11 mm^3^), a protein concentration of 20 mg/ml and tafamidis concentration of 678 μM in 10% D-DMSO (protein-to-ligand molar ratio of 1:2) were used (drop volume 40 µl; protein-to-buffer ratio of 1:1). For neutron data collection, the crystals were mounted in quartz capillaries and surrounded by a small amount of mother liquor from the crystallization well. The capillaries were sealed tightly using wax to eliminate the diffusion of gas and atmospheric water.

### Neutron data collection and processing

Details of the neutron data collection for WT-TTR have been reported by Haupt et al^49^. Neutron quasi-Laue diffraction data from the crystals of the S52P mutant and the S52P/tafamidis complex were collected at room temperature using the LADI-III diffractometer^50^ at the Institut Laue-Langevin (ILL), Grenoble. For the S52P mutant, a neutron wavelength range (Δλ/λ = 30%) of 2.9–3.9 Å was used, with data extending to 1.8 Å resolution. As is typical for a Laue experiment, the crystal was held stationary at different *φ* (vertical rotation axis) settings for each exposure. A total of 26 images (exposure time of 12 h per image) were collected from two different crystal orientations. For the S52P/tafamidis complex, a neutron wavelength range of 2.7–3.6 Å was used with data extending to 2.0 Å resolution. In total, 13 images were collected (exposure time of 8 h per image) from three different crystal orientations. The neutron diffraction images were indexed and integrated using the LAUE suite program *LAUEGEN*^51^. The program *LSCALE*^52^ was used to determine the wavelength-normalisation curve using the intensity of symmetry-equivalent reflections measured at different wavelengths and to apply wavelength-normalisation calculations to the observed data. The data were then merged in *SCALA*^53^. Relevant data collection statistics are summarised in Supplementary Table 2.

Neutron monochromatic diffraction data for the T119M mutant crystal were collected at room temperature using the instrument BIODIFF^54^ operated by the Forschungsreaktor München II (FRM II) and the Jülich Centre for Neutron Science (JCNS) at Garching. For data collection, the wavelength was set at 2.67 Å (Δλ/λ = 2.9%). A total of 202 frames were recorded with a rotation range of 0.35° and an exposure time of 53 min per frame. The diffraction data were indexed, integrated and scaled using *HKL2000*^55^ to a resolution of 1.85 Å. The output file of *HKL2000* in *SCA* format was converted into *MTZ* format by using *scalepack2mtz* program in the *CCP4* suite^56^. Relevant data collection statistics are summarised in Supplementary Table 2.

### X-ray data collection and processing

X-ray diffraction data of the crystals of all three variants were recorded on beamline ID30B^57^ at the European Synchrotron Research Facility (ESRF), Grenoble using a heavily attenuated X-ray beam (1.96% for S52P; 19.17% for T119M; 9.83% for S52P/tafamidis) of wavelength 0.9763 Å. For S52P and S52P/tafamidis complex, the same crystals used for data collection at LADI-III was used; whereas for T119M, a crystal from the same crystallization drop as the one used at BIODIFF was used. Data were recorded at room temperature on capillary-mounted crystals. Data were processed to the same maximum resolution of the corresponding neutron data with *XDS*^58^, scaled and merged with *SCALA*^53^, and converted to structure factors using *TRUNCATE* in the *CCP4* suite^56^. Relevant data collection statistics are summarised in Supplementary Table 2.

### Joint neutron and X-ray structure refinement

The PDB structures 5CLX (refined X-ray models of TTR S52P; data collection at 100 K) was used as starting models for joint X-ray and neutron refinement against the datasets of S52P and S52P/tafamidis; and 5CM1 (refined X-ray models of TTR T119M; data collection at 100 K) was used for the datasets of T119M. The *phenix.refine* program^59^ in the *PHENIX* package^60^ was used for refinement. The preparation of the starting model, the refinement settings and workflow, and the modelling of solvent molecules were as detailed in Haupt et al.^41^. Using the *ReadySet* option in *PHENIX*^60^, exchangeable hydrogen and deuterium atoms were placed at appropriate sites of the protein models with deuteriums elsewhere. For positions where both hydrogen and deuterium atoms were modelled, the occupancies of both were set to 0.5 and then their occupancies were refined, with their total occupancy constrained to 1. D_2_O molecules were added using *ReadySet* based on the positive neutron scattering length density in *F*_*o*_*–F*_*c*_ maps. *Coot*^61^ was used for model modifications, such as addition of solvent molecules, and rotamer- and torsion-angle adjustments, according to positive and negative nuclear scattering length density in both 2*F*_*o*_*–F*_*c*_ and *F*_*o*_*–F*_*c*_ maps. The final refinement statistics are summarised in Supplementary Table 3.

### Monitoring subunit composition of tetrameric  TTR by native MS

The details of the instrumental arrangements and sample preparation protocols have been described previously^18^. MS analyses were carried out on a quadrupole time-of-flight mass spectrometer (Q-TOF Ultima, Waters Corporation, Manchester, U.K.) that was modified for the detection of high masses^62,63^. As has been tested previously^18^, experiments carried out at 4 °C in static (non-shaking) conditions provided well-resolved MS spectra, hence these conditions were used for the work described here. Prior to native MS analysis, proteins were buffer-exchanged into 250 mM ammonium acetate pH 7. All the labile sites (i.e. N- and O-bound deuterium atoms) were allowed to exchange from D to H. The mass difference between (H)TTR and (D)TTR was contributed by carbon-bound Ds that were incorporated into the amino acid chain during synthesis and were non-exchangeable. Subunit exchange was initiated by mixing a solution containing one unlabelled protein variant and one with deuterium-labelled protein variant at 1:1 molar ratio. A concentration of 3 μM for each protein tetramer solution was used for all experiments. The relative abundance of the tetramers was calculated from the peak area of the 13 + to 15 + charge states and expressed as a percentage of the total area of the peaks assigned to the tetrameric TTR. For the experiment of mixing HS52P and DS52P in the presence of tafamidis, tafamidis was in 0.5% DMSO at a TTR:tafamidis molar ratio of 1:1. In this case, the relative abundance was calculated from the peak area of the 12 + to 14 + charge states. The presence of DMSO resulted in broader peaks on the MS spectra and less charge on the protein particles. The MS data were fitted by Bayesian regression using a first order kinetics model.

### Native MS data fitting and parameter estimation

Bayesian regression of the native MS data was performed in *python* using the *PyMC3* library^64^. The following one-phase exponential decay model was used:

1 $$y(t) = c + (y_0 - c)e^{ - kt}$$

where *t* is the time (in minutes), *y(t)* is the relative abundance of each species (in percentage), *y*_*0*_ is the abundance at *t* *=* *0*, *c* is the abundance at *t* *=* *∞* and *k* the rate constant (in inverse minutes); *c* *<* *y*_*0*_ for dissociating species, and *c* *>* *y*_*0*_ for associating species. For *y*_*0*_, a normally distributed prior was used, with mean 50% and standard deviation of 15% for dissociation, and with mean of 0% and standard deviation of 5% for association. For *c*, a uniform prior between 0% and *y*_*0*_ was used for dissociation, and between *y*_*0*_ and 100% for association. For *k*, a half normal prior with mean of 0 min^−1^ and standard deviation of 10 min^−1^ was used. White noise was modelled with a half normal prior with zero mean and standard deviation of 10%. The posterior estimate of the parameters was obtained by drawing 10,000 Markov chain Monte Carlo (MCMC) samples with the No-U-Turn Sampler (NUTS) algorithm. Five thousand tuning steps were carried out and discarded before colleting the posterior samples used for the estimate. The maximum a posteriori estimate (MAP) of the parameter space was passed to the sampler as the starting value. The mean and standard deviation of the rate constants (*k*) from their posterior distributions are reported.

### High-temperature molecular dynamics simulations

Molecular dynamics (MD) simulations were performed with Gromacs 2016^65^. The starting structure for the simulations was the WT (PDB-ID 4PVM) variant of TTR protonated at pH 7 with *pdb2gmx*. The dimeric and monomeric models were obtained from the same neutron crystal structure: for the dimers, chains A and B were used, and for the monomers chain A was used. The protein was modelled with the Amber99SB*-ILDNP^30,31^ and the Charmm36 (Nov. 2016)^32^ force field, and water molecules with the TIP3P model^66^. The protein was solvated in a dodecahedral box with periodic boundary conditions and a minimum distance between the solute and the box of 12 Å. Sodium and chloride ions were added to neutralise the systems at the concentration of 0.15 M.

Energy minimisation was performed using a steepest descent algorithm with force tolerance of 10 kJ mol^−1^ nm^−1^ for a maximum of 10,000 steps. 1 ns NVT and then NPT equilibrating simulations were performed with all solute heavy atoms restrained with a force constant of 1000 kJ mol^*−*1^ nm^*−*2^. In these simulations, the temperature was coupled with the stochastic v-rescale thermostat by Bussi et al.^67^. at the target temperature of 298 K; the pressure was controlled with the Berendsen weak coupling algorithm^68^ at a target pressure of 1 bar. A leap-frog integrator was used with a time-step of 4 fs and virtual sites^69^ were used for all solute hydrogens. All bonds were constrained with the P-LINCS algorithm^70^. The particle mesh Ewald (PME) algorithm^71^ was used for electrostatic interactions with a real space cut-off of 12 Å. The Verlet cut-off scheme was used with a Lennard–Jones interaction cut-off of 12 Å and a buffer tolerance of 0.005 kJ mol^−1^ ps^−1^. For the production simulations at 298 K, a single unbiased 1 μs simulation in the NPT ensemble was performed using the Parrinelo–Rahman pressure coupling scheme^72^. For the simulations at 598 K, ten simulations of 100 ns were performed in the NVT ensemble. Coordinates were saved every 50 ps.

The fraction of native contacts (*Q*), as described by Best et al.^33^, was employed as a measure of the degree of protein (un)folding and native-likeness of protein–protein interfaces. When used to describe protein folding, the list of native contacts was built by taking all pairs of heavy atoms *i* and *j* that are within 4.5 Å of each other and are on two different residues separated by at least three other residues. When describing protein–protein interfaces, the original definition of the authors was adapted so that the list of native contacts included all pairs of heavy atoms (*i, j*) that are within 4.5 Å of each other and are on two different protein chains. Once the set of atom pairs is defined, *Q* is then calculated as follows:

2 $$Q(x) = \frac{1}{N}\mathop {\sum}\nolimits_{(i,j) \in N} {\frac{1}{{1 + {\mathrm{exp}}[\beta (r_{i,j}\left( x \right) - \lambda r_{i,j}^0)]}}}$$

where *N* is the number of atom pairs (*i,j*) forming the native contacts, *r*_*i*_,_*j*_(*x*) is the distance between atom *i* and atom *j* in the configuration *x*, $r_{i,j}^0$ is the distance between *i* and *j* is the native state (i.e. the starting neutron crystal structure used), *β* is a smoothing parameters with value of 5 Å^−1^ and *λ* is a factor that accounts for fluctuations that takes the value of 1.8 for atomistic simulations. MD trajectories were analysed and *Q* calculated with scripts written in *python* and using the *mdtraj* library^73^.

Partial Least-Squares Functional Mode Analysis (PLS-FMA)^34,35^ was performed on the main chain of the proteins and using the fraction of native contacts as the functional property of interest. In particular, we analysed with PLS-FMA the trajectories of the tetramer simulations, using the fraction of native contacts of each monomeric unit as the measure of unfolding. About 75% of simulation snapshots were used for training and 25% for validation. The model was built using 20 PLS components, which resulted in a Pearson correlation coefficient between the data and the model ≥ 0.97 for both the training and the validation sets, for both force fields. We refer to the ensemble weighted maximally correlated mode (ewMCM) as described Krivobokova et al.^35^ simply as the PLS-FMA mode.

### Free energy calculations of protein mutation

Alchemical free energy calculations were performed using Gromacs 2016^65^ and a custom version of Gromacs 4.6 that implements the soft-core potential proposed by Gapsys et al.^74^. The input hybrid topologies were generated using the *pmx* python tool^75^. The starting structures for the calculations were the WT-TTR (PDB-ID 4PVM), S52P-TTR (PDB-ID 5NFW) and T119M-TTR (PDB-ID 5NFE) protonated at pH 7 with *pdb2gmx*. The dimeric and monomeric starting structures were obtained from the same neutron structures; for the dimers, chains A and B were used, and for the monomers chain A was used. The unfolded proteins were modelled as capped tripeptides, where the central amino acid was the one being mutated, and the other two were the adjacent amino acids in protein sequence. Proteins were modelled with the Amber99SB*-ILDNP force field^30,31^ and water molecules with the TIP3P model^66^. The proteins were solvated in a dodecahedral box with periodic boundary conditions and a minimum distance between the solute and the box of 12 Å. Sodium and chloride ions were added to neutralise the systems at the concentration of 0.15 M.

Energy minimisation was performed using a steepest descent algorithm for a maximum of 10,000 steps. Temperature and pressure were equilibrated with ten independent 0.5 ns simulations in the NPT ensemble, with all solute heavy atoms restrained with a force constant of 1000 kJ mol^*−*1^ nm^*−*2^. A leap-frog stochastic dynamics integrator^76^ was used with time-step of 2 fs; temperature was coupled using Langevin dynamics at the target temperature of 298 K, and pressure with the Berendsen weak coupling algorithm^68^ at a target pressure of 1 bar. The particle mesh Ewald (PME) algorithm^71^ was used for electrostatic interactions with a real space cut-off of 11 Å, a spline order of 4, a relative tolerance of 10^*−*5^ and a Fourier spacing of 1.2 Å. The Verlet cut-off scheme was used with a Van der Waals interaction cut-off of 11 Å and a buffer tolerance of 0.005 kJ mol^−1^ ps^−1^. Bonds involving hydrogens were constrained with the P-LINCS algorithm^70^. Ten equilibrium simulations of 10 ns duration were then initiated from the last frame of the short (0.5 ns) equilibration simulations. From each of the ten equilibrium simulations, a non-equilibrium trajectory was spawn every 0.2 ns, for a total 50 trajectories per equilibrium simulation and 500 trajectories overall. Forward and reverse transformations were performed for both mutants, with 500 non-equilibrium trajectories per transformation: WT→S52P, S52P→WT, WT→T119M, T119M→WT. Coordinates for hybrid residues were built with *pmx*^75^ after extracting the starting configurations of the systems. Energy minimisation was performed on the dummy atoms only, before equilibrating velocities with a 10 ps simulation. Then, the non-equilibrium alchemical transformation was performed over 100 ps. The non-equilibrium simulations were performed using a custom version of Gromacs 4.6 that implements the soft-core potential described in Gapsys et al.^74^, where both vdW and Coulombic interactions were soft-cored. Free energy differences were estimated using the Bennet’s Acceptance Ratio (BAR)^77^ as implemented in *pmx*^75^. Uncertainties in the *ΔG* values were calculated by taking the standard error of the BAR estimate from the ten independent equilibrium simulations and related non-equilibrium trajectories.

### Grand canonical monte carlo calculations

The simulation package ProtoMS 3.3 was used for Grand Canonical Monte Carlo (GCMC) calculations and data analysis^29,78^. The starting tetramer structures for the calculations were the WT-TTR (PDB-ID 4PVM), S52P-TTR (PDB-ID 5NFW), S52P-TTR/tafamidis (PDB-ID 6FFT) and T119M-TTR (PDB-ID 5NFE) with protonation states as resolved experimentally. Proteins were modelled with the Amber ff14SB force field^79^, water with the TIP3P model, and tafamidis with the GAFF/AM1-BCC force field^66,80^.

For the calculation of the stability of W17, a GC box was created around the water molecule by adding 2 Å padding in all three dimensions and used for both the calculations in S52P-TTR and S52P-TTR/tafamidis. For the calculation of the monomer–monomer interface hydration free energy, the grand canonical box was defined so to encompass the interface containing the conserved water molecules of interest; the same box was used for all proteins, which had been previously aligned. Because the hydration free energy results might be sensitive to the protonation state of the histidine residues in proximity of these conserved water molecules, from each experimental structure we generated the other two proteins by mutating the amino acids at positions 52 and 119. Thus, for each TTR variant, we performed three sets of calculations, based on each neutron structure and its respective protonation states of His31, His56, His88 and His90.

Protein residues that were further than between 16 and 20 Å away from the grand canonical region were removed, with the exact distance chosen to retain whole residues. The systems were then solvated up to a radius of 30 Å around the grand canonical region. All simulations were carried out at 298 K, and a 10 Å cut off was applied to the non-bonded interactions. Before initiating the GCMC simulations, the systems were equilibrated using 50 million (M) solvent-only moves in the canonical ensemble, so to equilibrate the water around the proteins. Water molecules present in the pre-defined GCMC box were then removed; this set of coordinates represented the starting point of the GCMC simulations.

For the calculation of the monomer–monomer interface hydration, a set of 32 simulations were performed at a range of Adams values from −29 to + 2 at unit increments. For the calculations of the stability of W17, Adams values between −18 and 0 were used instead. For each window, 15 M equilibration moves were performed only on the grand canonical solute, with insertion, deletion and translation/rotation moves generated at the same ratio. An additional 5 M equilibration moves followed where the protein and solvent molecules were also sampled, before starting the production simulation of 50 M moves. Half of the MC moves were dedicated to the grand canonical water molecules, and the other half was split between protein residues and solvent in proportion to the number of solvent molecules and protein residues, according to the ratio of 1:5. Hamiltonian exchange was employed^78^, with exchanges being performed every 0.2 M steps. Data for analysis, energies, number of GC solutes present, and coordinates were saved to file every 0.1 M moves.

The above procedure was repeated three times for the monomer–monomer interface hydration calculation (i.e. 9 calculations for each TTR variant, three repeated calculations per starting structure) and ten times for the W17 stability calculation. The hydration free energy of the whole grand canonical region, and the binding free energy of individual water molecules, were computed using *grand canonical integration* (GCI) as described in Ross et al.^29^. GCI was performed using the *calc_gci.py* script that is part of the ProtoMS 3.3 tools, with the data from all repeats being analysed together. The amount of data discarded as equilibration was determined using the equilibration detection tool available in *calc_series.py*. The same amount of data was discarded from all windows after determining the average number of moves needed for equilibration.

## Supplementary information

Supplementary Information

Reporting Summary

## Data Availability

Atomic coordinates and diffraction data have been deposited in the Protein Data Bank (accession codes 5NFW for S52P-TTR, 5NFE for T119M-TTR, and 6FFT for S52P-TTR/tafamidis complex). Other data are available from the corresponding author upon reasonable request.
