# Supplementary material for: A molecular mechanism for transthyretin amyloidogenesis
Source: Nat Commun. 2019 Feb 25;10:925. doi: 10.1038/s41467-019-08609-z (PMC6390107; doi:10.1038/s41467-019-08609-z)
Supplement: Supplementary file 1 — Supplementary Information [file 41467_2019_8609_MOESM1_ESM.pdf]

Supplementary Information

A molecular mechanism for transthyretin amyloidogenesis

Ai Woon Yee, Matteo Aldeghi *et al.*

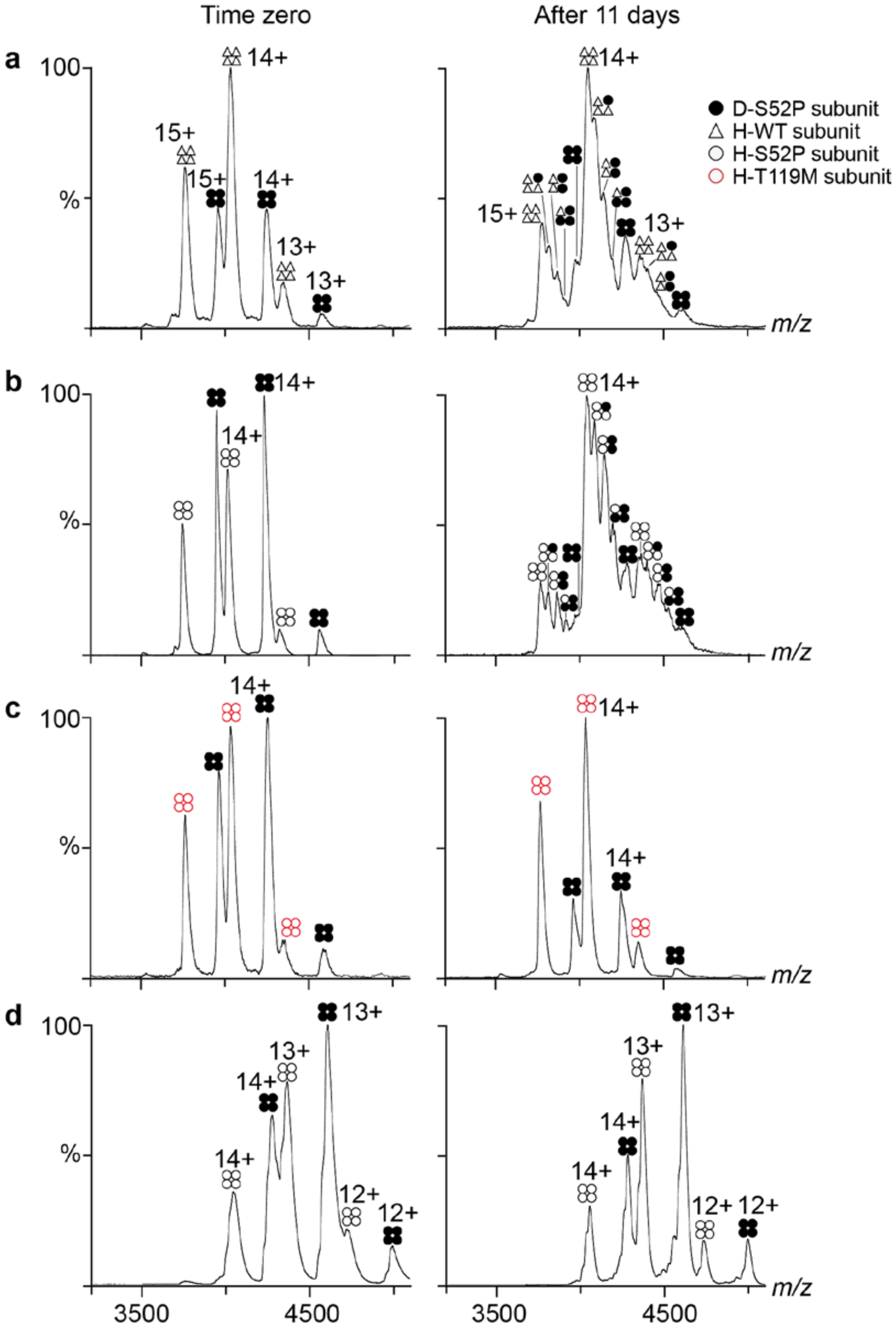

Supplementary Figure 1. Native mass spectrometry spectra of the subunit exchange experiments between deuterated S52P and hydrogenated (**a**) WT, (**b**) S52P, (**c**) T119M, and (**d**) S52P bound to tafamidis, respectively. A concentration of 3 μM for each protein tetramer was used for all experiments. The spectra shown were recorded at the beginning of the reaction (*left panel*) and after 11 days (*right panel*). It should be noted that DMSO, in which tafamidis was dissolved, reduced the charge states of TTR.

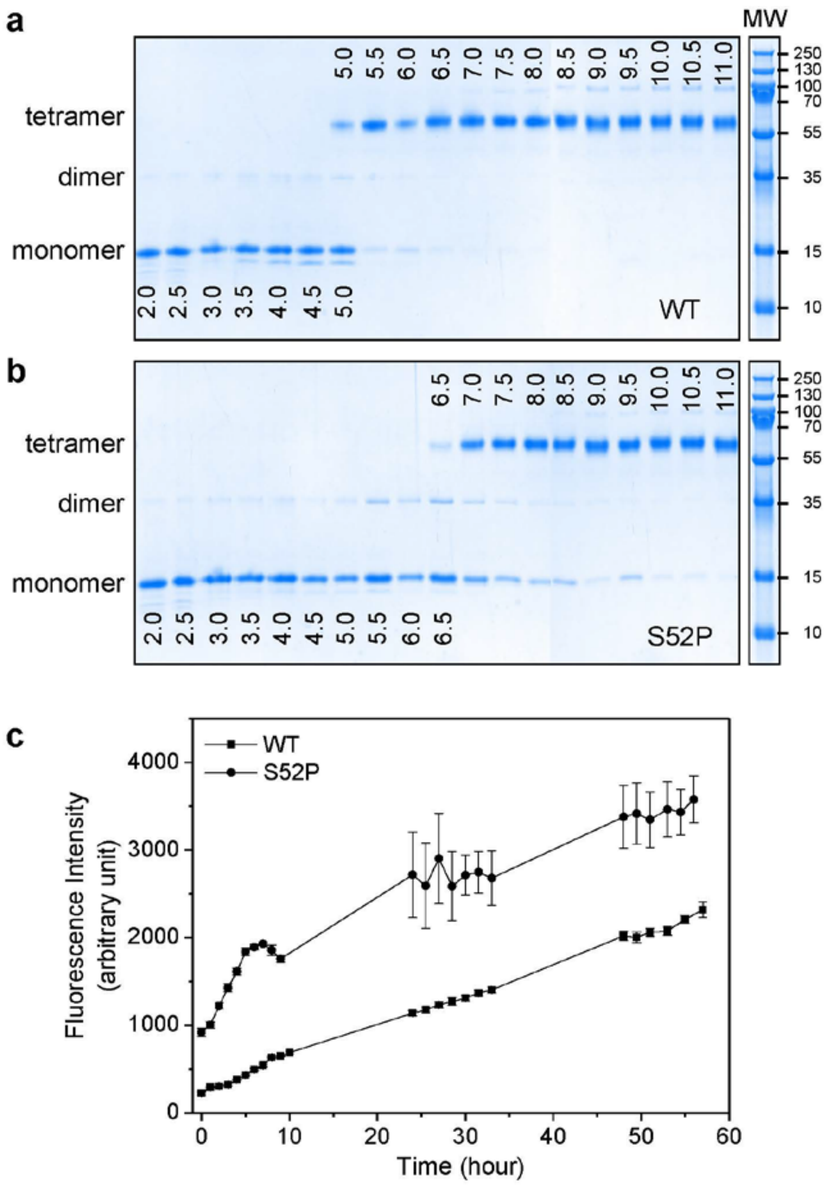

Supplementary Figure 2. Comparison of tetramer stability and amyloid fibrillation between WT-TTR and TTR S52P mutant. (**a-b**) WT and S52P tetramer stability as a function of pH shown on Tris-Tricine Coomassie-stained gel. This method provides a simple evaluation of the quaternary structure stability, on the basis that a stable protein withstands acidic buffers without undergoing dissociation.[1,2] Bands corresponding to S52P monomers can be seen between pH 5.5 and 11, implying huge amount of tetrameric dissociation to monomers; these bands are absent for the WT. Each panel is a composite of two gels. The pH value is indicated on each lane. Molecular weight markers are shown on the right in kDa. Three major bands were detected, corresponding to TTR tetramer (~56 kDa), dimer (~28 kDa), and monomer (~14 kDa). (**c**) ThT fluorescence intensities measured during the fibrillation of WT and S52P (λ_{ex} = 440 nm, λ_{em} = 480 nm). Higher ThT fluorescence indicates more amyloid fibril binding.[3] Each data point is the average of triplicate measurements; error bars are the standard deviations.

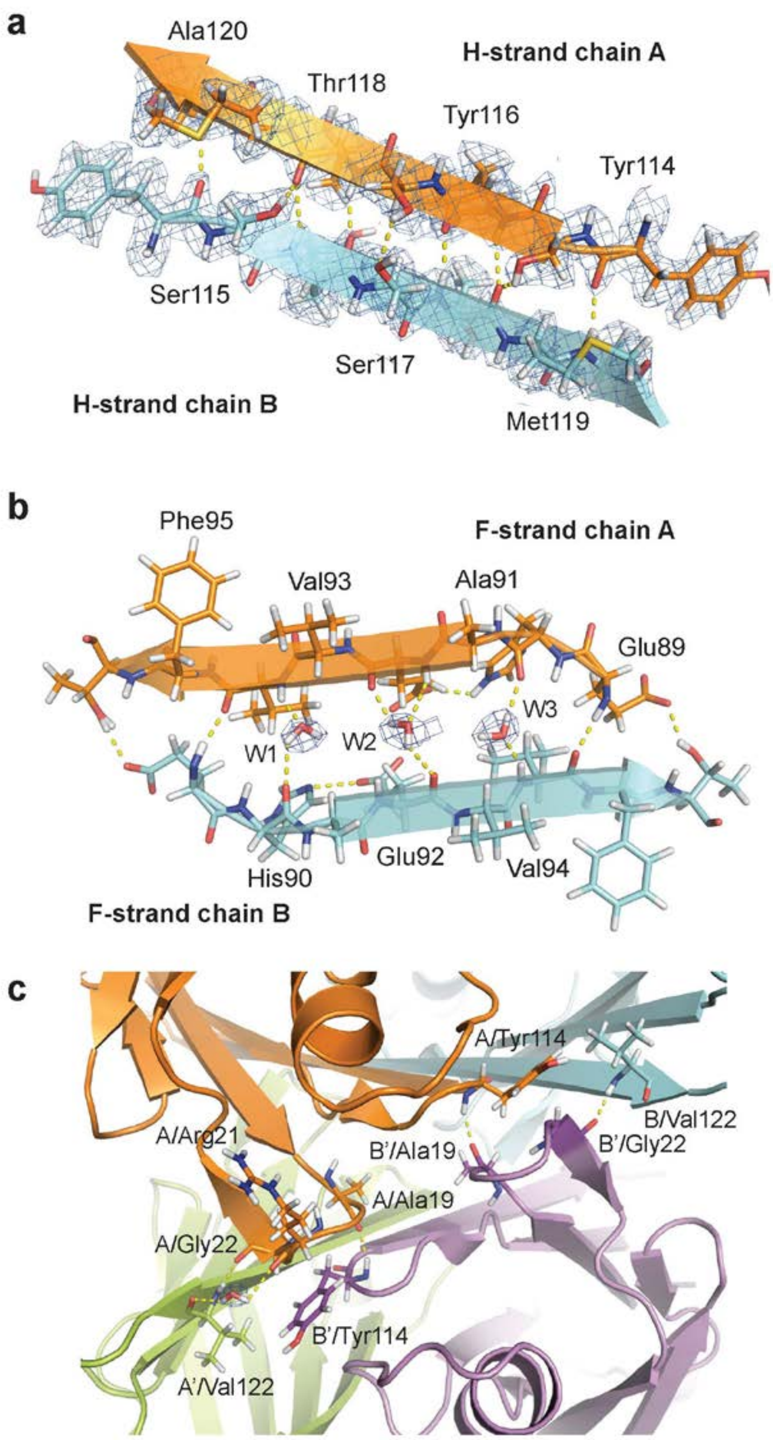

Supplementary Figure 3. Hydrogen bonds and water molecules involved at the monomer-monomer interface (**a-b**) and dimer-dimer interface (**c**) of the TTR T119M structure. In (**c**), four of the eight contacts are shown with a dashed line. One water molecule forms hydrogen bonds to A'/Val122/O and A/Arg21/O. The other four hydrogen bonds and another water molecule, not visible in the picture, are the equivalent paired residues on the reverse side of the protein. Blue mesh is the $2F_o$–F_c neutron scattering length density map contoured at 1.5σ.

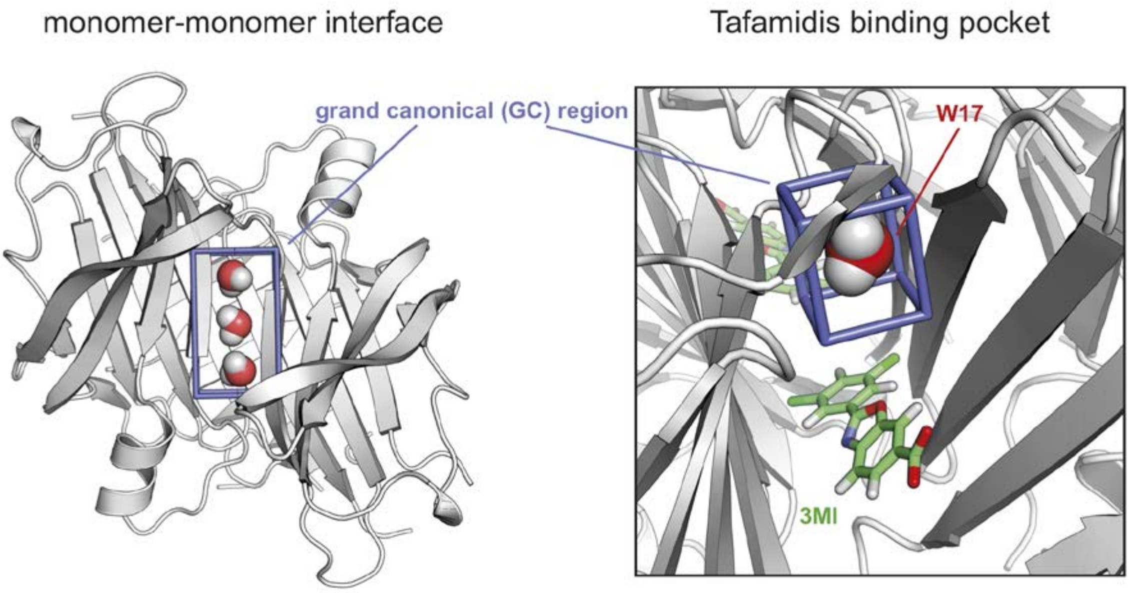

Hydration free energy of the interface (kcal mol⁻¹)

WT	S52P	T119M	Average
−20.8 ± 2.2	−20.3 ± 1.4	−20.3 ± 1.9	−20.5 ± 0.2

W17 stabilisation upon tafamidis binding (kcal mol⁻¹)

S52P	S52P/tafamidis	W17 stabilisation
−0.8 ± 0.1	−2.2 ± 0.0	−1.4 ± 0.1

Supplementary Figure 4. Water binding free energies as calculated with Grand Canonical Monte Carlo simulations. At the monomer-monomer interface, water molecules were estimated to be tightly bound (with hydration free energy of about -20 kcal/mol), thus having a highly stabilising effect on the monomer-monomer interface. There are no significant differences between the three TTR variants. These free energy values are the mean and standard error obtained from three calculations that were started from three different joint neutron/X-ray crystal structures (see Methods). For the stabilization free energy of W17, the hydration free energies of this water molecules in the complex (S52P/tafamidis) and *apo* protein (S52P) were calculated and then subtracted. Each molecule of tafamidis binding affects two such water molecules due to the intrinsic 2-fold crystallographic symmetry. In this case, the free energy estimates are the mean and standard error obtained from ten calculations.

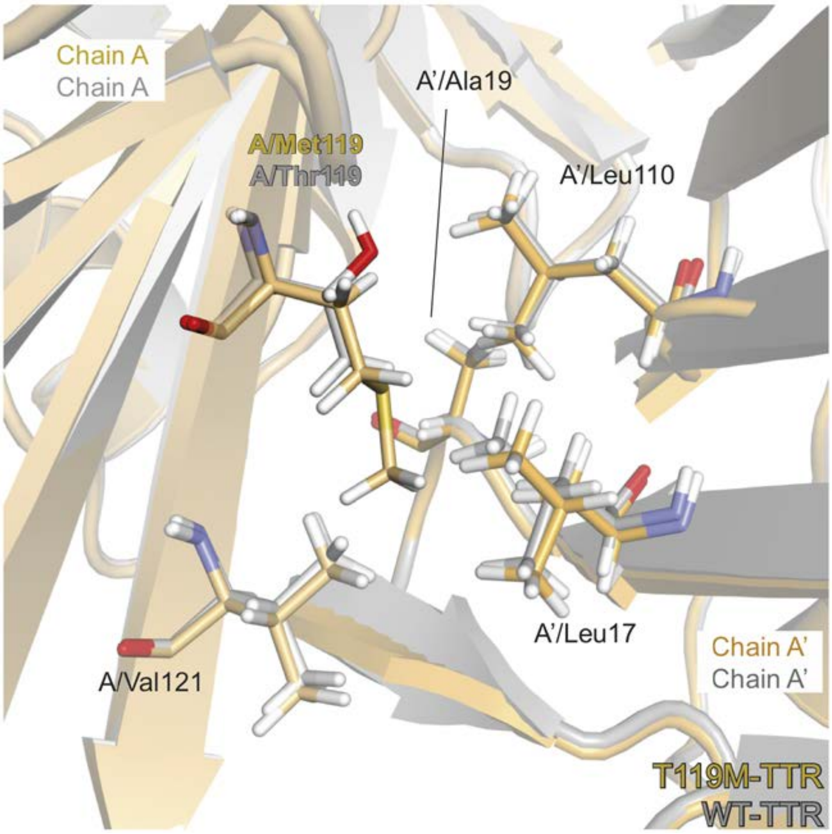

Supplementary Figure 5. Comparison of residue 119 in WT-TTR (*grey*) and TTR-T119M (*gold*). In the T119M mutant, the Met119 residue has a longer side chain which extends across the thyroxine-binding channel into a hydrophobic pocket surrounded by residues Leu17, Ala19, Leu110 and Val121. Such interactions substantially enhance the association of two dimers and hence the overall stability of the tetramer.

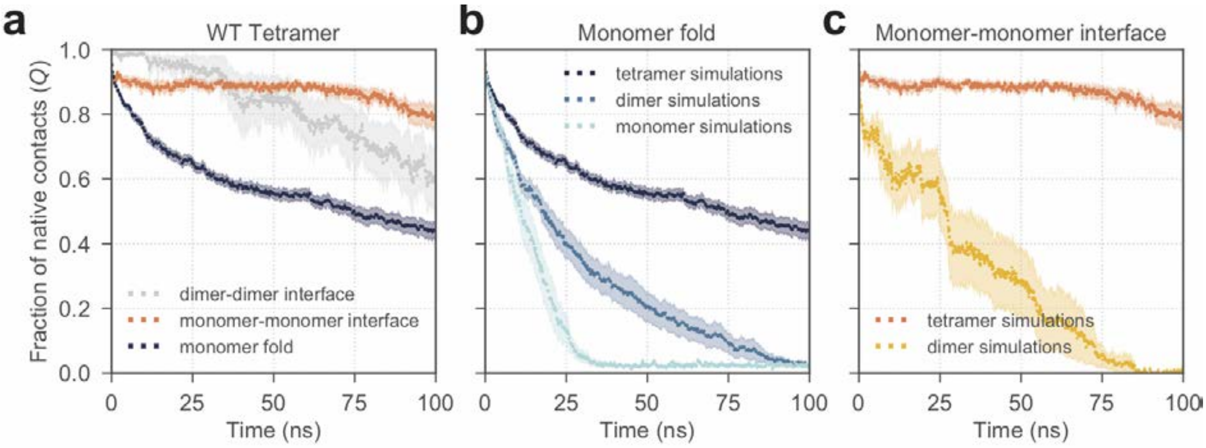

Supplementary Figure 6. Loss of native fold and contacts in WT-TTR during high-temperature MD simulations with the Charmm36 force field; shown are the mean and its standard error from 10 simulation repeats. (**a**) Degree of disruption of the WT-TTR protein fold, monomer-monomer, and dimer-dimer interface monomers, when simulating the WT tetramer over the course of 100 ns. (**b**) Degree of unfolding of WT-TTR monomers as part of a tetramer, dimer, or monomer in solution. (**c**) Degree of disruption of the WT-TTR monomer-monomer interface when simulating the dimer and the tetramer.

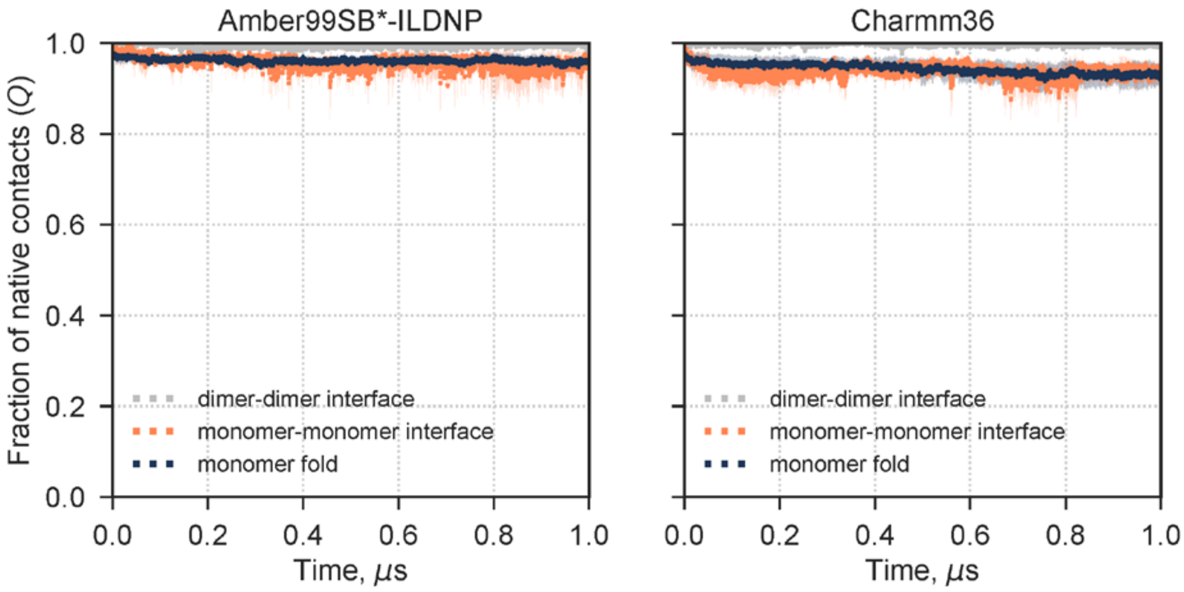

Supplementary Figure 7. Fraction of native contacts (Q) observed during simulations at 298K for TTR-WT. The monomer fold, and the monomer-monomer and the dimer-dimer interfaces are stable during the 1 μs simulation in both the Amber and Charmm force fields.

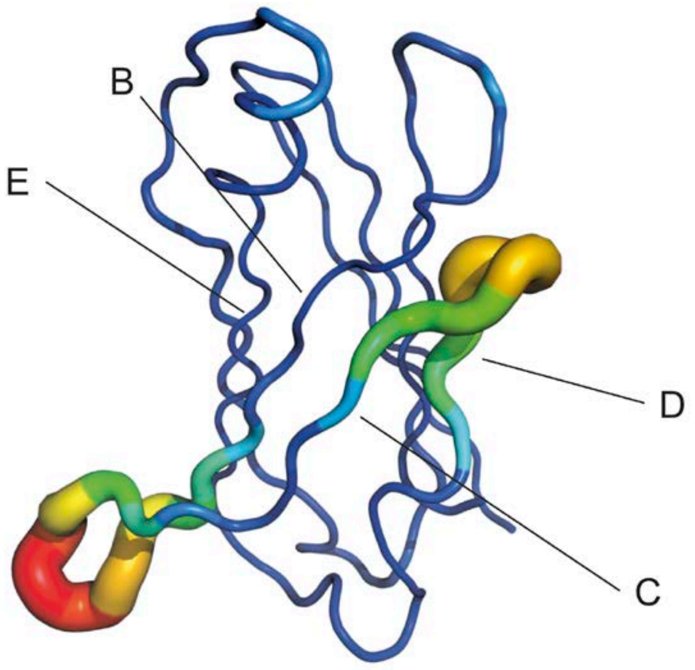

Supplementary Figure 8. Protein regions in the PLS-FMA mode contributing most to the change in the fraction of native contacts for the high-temperature simulations in Charmm36.

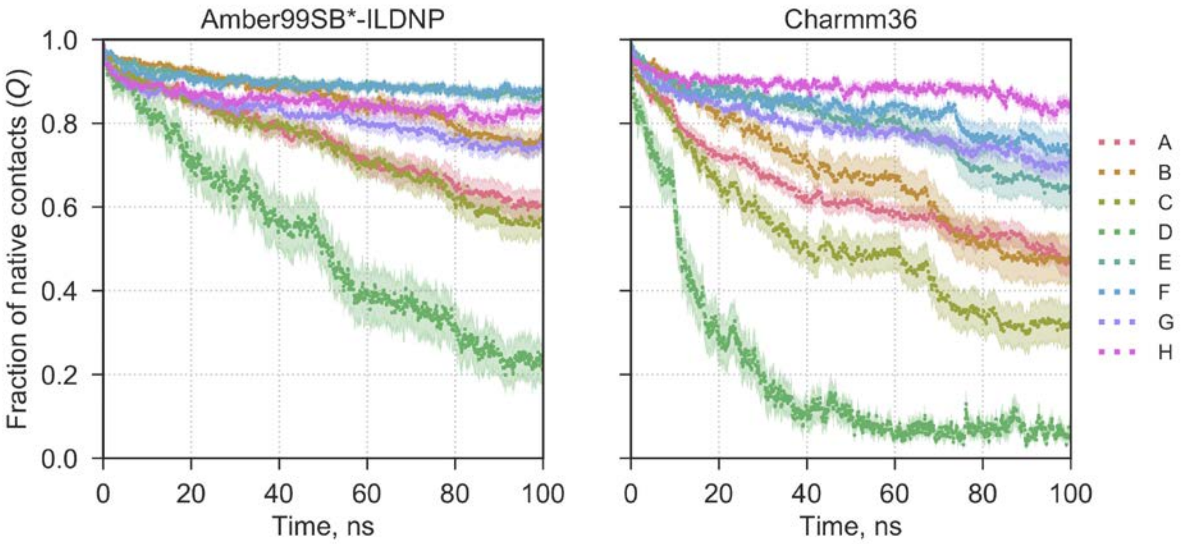

Supplementary Figure 9. Degree of unfolding by strand over the course of 100 ns simulations, as measured by the fraction of native contacts (*Q*) for WT tetramers in solution. Shown are the mean and its standard error from 10 simulation repeats.

**WT-TTR cDNA sequences**	ATGGGTCCTA CGGGCACCGG TGAATCCAAG TGTCCTCTGA TGGTCAAAGT TCTAGATGCT GTCCGAGGCA GTCCTGCCAT CAATGTGGCC GTGCATGTGT TCAGAAAGGC TGCTGATGAC ACCTGGGAGC CATTTGCCTC TGGGAAAACC AGTGAGTCTG GAGAGCTGCA TGGGCTCACA ACTGAGGAGG AATTTGTAGA AGGGATATAC AAAGTGGAAA TAGACACCAA ATCTTACTGG AAGGCACTTG GCATCTCCCC ATTCCATGAG CATGCAGAGG TGGTATTCAC AGCCAACGAC TCCGGCCCCC GCCGCTACAC CATTGCCGCC CTGCTGAGCC CCTACTCCTA TTCCACCACG GCTGTCGTCA CCAATCCCAA GGAATGA
**S52P mutation (t157c) forward primer**	5'-gggaaaaccagtgagcctggagagctgcatg-3'
**S52P mutation (t157c) reverse primer**	5'-catgcagctctccaggctcactggttttccc-3'
**T119M mutation (c359t) forward primer**	5'-gtgacgacagccatggtggaataggagtaggg-3'
**T119M mutation (c359t) reverse primer**	5'-ccctactcctattccaccatggctgtcgtcac-3'

Supplementary Table 1. WT-TTR cDNA sequence and the sequences of primers used for S52P and T119M mutations are shown.

	S52P (N)	S52P (X)	T119M (N)	T119M (X)	S52P/tafamidis (N)	S52P7tafamidis (X)
**Data collection**						
Space group			$P2_12_12$			
Cell dimensions						
a, *b*, *c* (Å)	43.78, 86.30, 65.53†		43.71, 86.32, 65.34	43.61, 86.20, 65.18	43.74, 85.82, 65.27	43.90, 85.70, 65.54
α, β, γ (°)	90.00, 90.00, 90.00		90.00, 90.00, 90.00	90.00, 90.00, 90.00	90.00, 90.00, 90.00	90.00, 90.00, 90.00
Resolution (Å)	33.54-1.80 (1.90-1.80)	43.78-1.80 (1.90-1.80)	33.49-1.85 (1.90-1.85)	43.61-1.85 (1.95-1.85)	35.87-2.00 (2.11-2.00)	43.90-2.00 (2.11-2.00)
R_{merge}	0.159 (0.202)	0.048 (0.070)	0.164 (0.526)	0.047 (0.062)	0.121 (0.208)	0.041 (0.046)
I / σI	8.4 (4.9)	33.4 (20.5)	3.7 (1.2)	27.1 (16.7)	6.0 (4.0)	31.0 (23.3)
Completeness (%)	79.5 (54.3)	98.7 (99.5)	90.6 (83.7)	97.3 (99.1)	71.9 (56.3)	98.7 (99.5)
Redundancy	5.2 (2.8)	6.5 (6.6)	2.0 (1.7)	4.4 (4.3)	2.6 (2.2)	4.6 (4.7)

*Values in parentheses are for highest-resolution shell.
†X-ray cell dimensions are used for the neutron structure.

Supplementary Table 2. X-ray and neutron data collection statistics of the crystal structures of S52P, T119M, and S52P/tafamidis complex (N=neutron; X=X-ray).

**Refinement**	**S52P**	**T119M**	**S52P/tafamidis**
Resolution (Å)	N: 33.54–1.80 (1.85–1.80)<br>X: 39.04–1.80 (1.84–1.80)	N: 33.49–1.85 (2.00–1.85)<br>X: 43.16–1.85 (1.90–1.85)	N: 35.87–2.00 (2.08–2.00)<br>X: 42.85–2.00 (2.06–2.00)
No. reflections N	18453 (795)	19744 (1151)	12097 (864)
No. reflections X	23338 (1238)	20896 (1237)	17192 (1254)
R_{work} / R_{free} N	0.2114 / 0.2607	0.2132 / 0.2536	0.2313 / 0.2801
R_{work} / R_{free} X	0.1724 / 0.2103	0.1648 / 0.1942	0.1574 / 0.1947
No. atoms			
Protein	3893	3931	3895
Ligand/ion	-	-	52
Water	105	100	77
No. D_2O, D–O, O-only molecules	30, 4, 7	28, 6, 4	25, 1, 0
B-factors			
Protein chain A	33.02	31.02	32.67
Protein chain B	39.39	37.89	40.81
Ligand/ion	-	-	32.21
Water	36.09	33.74	32.77
R.m.s. deviations			
Bond lengths (Å)	0.015	0.014	0.017
Bond angles (°)	1.63	1.55	1.98
Ramachandran statistics (%)			
Favoured	96.5	97.4	96.1
Allowed	3.1	2.6	3.5
Outliers	0.4	0.0	0.4

*Values in parentheses are for highest-resolution shell.

Supplementary Table 3. Joint X-ray and neutron refinement statistics of the crystal structures of S52P, T119M, and S52P/tafamidis complex.

Supplementary References:

1. Lai, Z., Colón, W. & Kelly, J. W. The acid-mediated denaturation pathway of transthyretin yields a conformational intermediate that can self-assemble into amyloid. *Biochemistry* **35,** 6470–82 (1996).
2. Lundberg, E., Olofsson, A., Westermark, G. T. & Sauer-Eriksson, a E. Stability and fibril formation properties of human and fish transthyretin, and of the Escherichia coli transthyretin-related protein. *FEBS J.* **276,** 1999–2011 (2009).
3. Khurana, R. *et al.* Mechanism of thioflavin T binding to amyloid fibrils. *J. Struct. Biol.* **151,** 229–238 (2005).
